# Risk factors for acute toxoplasmosis in the Netherlands

**DOI:** 10.1017/S0950268823000808

**Published:** 2023-05-24

**Authors:** Ingrid H. M. Friesema, Agnetha Hofhuis, Denise Hoek-van Deursen, Arjan R. Jansz, Alewijn Ott, Jaap J. van Hellemond, Joke van der Giessen, Laetitia M. Kortbeek, Marieke Opsteegh

**Affiliations:** 1 Centre for Infectious Disease Control, National Institute for Public Health and the Environment (RIVM), Bilthoven, The Netherlands; 2 Department of Medical Microbiology, Eurofins-PAMM, Veldhoven, The Netherlands; 3 Department of Medical Microbiology, Certe, Groningen, The Netherlands; 4 Department of Medical Microbiology & Infectious Diseases, Erasmus MC University Medical Centre, Rotterdam, The Netherlands

**Keywords:** Case-control study, risk factors, *Toxoplasma gondii*, toxoplasmosis

## Abstract

Toxoplasmosis caused by the protozoan parasite *Toxoplasma gondii* occurs worldwide. Infections range from asymptomatic to life-threatening. *T. gondii* infection is acquired either via bradyzoites in meat or via oocysts in the environment, but the relative importance of these path ways and the different sources remains unclear. In this study, possible risk factors for toxoplasmosis in the Netherlands were investigated. A case–control study was conducted including persons with recent infection and individuals with a negative test result for IgM and IgG for *T. gondii* between July 2016 and April 2021. A total of 48 cases and 50 controls completed the questionnaire. Food history and environmental exposure were compared using logistic regression. Consumption of different meats was found to be associated with recent infection. In the multivariable model, adjusted for age, gender, and pregnancy, consumption of large game meat (adjusted odds ratio (aOR) 8.2, 95% confidence interval 1.6–41.9) and sometimes (aOR 4.1, 1.1–15.3) or never (aOR 15.9, 2.2–115.5) washing hands before food preparation remained. These results emphasize the value of the advice to be careful with the consumption of raw and undercooked meat. Good hand hygiene could also be promoted in the prevention of *T. gondii* infection.

## Introduction

The protozoan parasite *Toxoplasma gondii* is the cause of toxoplasmosis in humans. Both the parasite and the disease are spread worldwide. About one-third of the human population has been infected with the parasite, whereby the majority of the infections is asymptomatic or otherwise mild and self-limited [1, 2]. Congenital-infected children and immunocompromised people are at the highest risk of severe clinical manifestations. Primary infection during pregnancy can cause congenital infection that may lead to miscarriage, stillbirth or prematurity, and a wide spectrum of clinical manifestations in the newborn [2]. In Europe, where *T. gondii* genotype II is predominant, 75% of children with congenital infection are asymptomatic at birth; however, if left untreated, these children may develop symptoms at a later stage in life [3]. At birth, a minority of the congenitally infected children have manifestations such as chorioretinitis, intracranial calcifications, or hydrocephalus [2, 3]. Severe toxoplasmosis in immunocompromised children and adults mainly manifests central nervous system disease, myocarditis, or pneumonitis [2, 4].

Cats, as definitive hosts, are essential within the life cycle of *T. gondii* as they shed oocysts in the environment. Ingestion of tissue cysts or oocysts by a cat can lead to the subsequent excretion of millions of oocysts [5]. Sporulated oocysts can survive for long periods in the environment and can end up in water, on fresh produce, or ingested by wildlife or livestock [5, 6]. Homeothermic vertebrates, other than felines, do not shed oocysts, but in these intermediate hosts, intracellular tissue cysts develop and may remain in the tissue for life. These tissue cysts are also infectious to definitive and intermediate hosts, for example, through the consumption of raw or undercooked meat of animals infected with *T. gondii.* Transmission of *T. gondii* to humans can occur by ingestion of sporulated oocysts in soil, food, or water or by direct contact with the feces of infected cats that shed oocysts, or by consumption of undercooked or raw meat containing tissue cysts [4, 5]. Moreover, *T. gondii* can be transmitted vertically during pregnancy, or through blood transfusions or organ transplant [4, 5]. Cultural habits, such as food and hygiene practices, influence the relative contribution of different modes of transmission to the incidence of toxoplasmosis.

In the Netherlands, the seroprevalence of *T. gondii* in the general population was estimated in the PIENTER-study; serological surveys were conducted in 1996/1997 (40.5%) [7], 2006/2007 (26.0%) [8], and 2016/2017 (29.9%) [unpublished observations]. Identified risk factors differed between surveys and included increasing age, certain regions in the Netherlands, relatively low education level, cat ownership, gardening, and consumption of raw meat, especially raw/undercooked pork. Presence of *T. gondii* DNA in soil was examined in a study performed in the Netherlands between August 2018 and November 2019 [9]. A total of 5 out of 148 soil samples (3%, 95% confidence interval (CI): 1.5–7.7%) were positive. Most of the examined samples and all five samples that tested positive were from private backyard gardens, and the rest were from playgrounds. The main source of human infection remained unclear in the Netherlands: infection by consumption of meat containing bradyzoites or by oral ingestion of sporulated oocysts present in the environment.

A meta-analysis of 187 studies worldwide revealed environmental factors such as contact with soil or cats and consumption of raw or undercooked meat, unwashed vegetables, shellfish, and raw milk are significant risk factors for toxoplasmosis [10]. However, it is known that risk factors may differ between countries. Moreover, these are factors associated with *T. gondii* infections without considering the moment of infection. Since the antibody response usually remains detectable lifelong, the infection may have been acquired long before taking the questionnaire, possibly resulting in misclassification of exposure. Studies analyzing risk factors for recently acquired *T. gondii* infection in the general population are scarce. The main risk factor in these studies was consumption of raw or undercooked meat [11–16]. Consumption of other foods identified as risk factors in these studies were unpasteurized goat milk, shellfish, and unwashed raw vegetables or fruits. Environmental factors identified in these studies were having a pet cat or kittens, cleaning the cat litter box, or contact with soil [12–14, 16]. In the present case–control study, risk factors for recently acquired *T. gondii* infection in the Netherlands were investigated.

## Methods

### Study population

The study population consisted of individuals who were tested for toxoplasmosis by serological methods between 15 July 2016 and 30 April 2021 at one of the 14 participating regional medical microbiology laboratories in the Netherlands. Six laboratories were located in the west (provinces of Noord Holland, Zuid Holland, Utrecht), two in the north (Friesland, Groningen, Drenthe), two in the east (Overijssel, Gelderland) and four in the south (Noord Brabant, Limburg). A case was defined as a patient with acute toxoplasmosis if tested positive for *T. gondii* specific IgM in combination with a low to intermediate avidity (<60%) of *T. gondii* specific IgG antibodies. IgG avidity is a measure of the binding strength of IgG antibodies to an antigen [17]. Avidity increases over time as prolonged or repeated exposure to the antigen results in the production of *T. gondii* specific IgG, which binds more strongly to the antigens. Controls in this study were individuals with a negative test result for both IgM and IgG to *T. gondii.*

The regional microbiology laboratories were asked to send a letter to the treating physician with the test result, an explanation of the study, and an envelope with study materials for the acute toxoplasmosis case. The physician was then asked to forward the study materials (an explanation of the study for the case, an informed consent form, and a questionnaire) to the case, after which the case could send the completed questionnaire with informed consent form to the National Institute for Public Health and the Environment (RIVM). Furthermore, the regional laboratories were asked to select three individuals who tested negative within the same testing period (+/− 2 months) and matched for age (maximum difference 10 years) and gender. If the case was pregnant, the selected controls also had to be pregnant. The controls received their study material in the same way as the cases. No ethical approval was required to conduct this study, as decided by the Medical Research Ethics Committee (METC-protocol number 16-384/C).

### Questionnaire

The questionnaire (61 questions) covered six topics: A) Personal data: birth date, gender, body length and weight, postal code, country of birth, and education level (12 questions); B) Health and pregnancy: reasons for testing, pregnancy, medical conditions and diseases, medication use, symptoms, and foreign travel (eight questions); C) Food and eating habits: consumption of meat, including by species, specific meat products and whether eaten raw or undercooked, freezer storage, consumption of other animal products (i.e., eggs, dairy products) and raw vegetables, fruits, and herbs (17 questions); D) Kitchen hygiene: hand washing, use and cleaning of cutting boards and knives (five questions); E) Contact with cats and cat feces: owning or caring for a cat, number of cats, indoor/outdoor cats, using and cleaning of litter box (10 questions); F) Contact with animals, soil, sand, and water: occupation, contact with animals as part of occupation or volunteer work, visiting a farm, outdoor activities, gardening, contact with soil, mud, sand, or surface water (nine questions).

The reference period for the questions was 9 months before the test, except for the questions on personal data and reasons for testing. Although high IgG avidity is considered indicative of infection at least 4 months ago, antibody maturation is variable, and persistently low avidity values have been reported [17]. Since our aim was to capture all behavior that may have led to the infections identified in the current study based on the presence of IgM and IgG with low or intermediate avidity, it was decided to use a longer reference period than the 4 months associated with high avidity.

### Serological examination

The laboratories were asked to send the serum of cases to RIVM for serological examination to confirm that the case met the criteria for acute toxoplasmosis and to exclude variation in results due to different serological methods. IgG antibodies to *T. gondii* were determined using an in-house sandwich ELISA at a serum dilution of 1:20 (adapted from the method described by Ruitenberg and van Knapen [18]). In short, the antigen used in this ELISA was a saponin-octylglucoside solubilized fraction prepared from *T. gondii*-RH tachyzoites cultured in vitro, and the conjugate was a commercially available peroxidase-labeled antihuman IgG conjugate (Dako, Glostrup, Denmark). Patient sera were examined in a dilution series starting at 1:20 and compared with a cut-off pooled serum standard that was prepared and lyophilized in 1978 and has been used ever since. For this study, only the qualitative results of the ELISA were used (positive or negative in the case of an optical density result above or below the cut-off serum standard, respectively). IgM antibodies to *T. gondii* were determined using a commercial agglutination assay (Toxo ISAGA IgM Kit bioMérieux 75361) [19, 20]. The avidity was determined using a commercial ELISA (*T. gondii* avidity determination, Euroimmun EL2410-9601-1G) [17].

### Data analyses

Data were analyzed using SAS software, version 9.4 M7 (SAS Institute Inc., USA). Demographic characteristics between responding and nonresponding cases were tested using chi-square tests, as were demographic characteristics, reason for testing, and symptoms between participating cases and controls. Exposures mentioned by at least 20% of cases were analyzed using logistic regression. First, univariable analysis was performed with calculation of odds ratios (ORs) and 95% confidence intervals (95% CI). Factors with *P* < 0.15 in the univariable analysis were included in the multivariable model, and adjusted for age (18–29, 30–39, 40–49, >50 years), gender, and pregnancy. Due to the relatively small number of participants and as consumption of meat and consumption of raw or undercooked meat partly overlap, not all identified variables could be included in a multivariable model. Calculation of correlations among variables showed correlation coefficients above 0.60 only among meat products; therefore, beef and pork were included only as consumption of raw and undercooked beef and pork, respectively, and not as the different specific meat products. A final model was determined by stepwise elimination of variables, but always adjusted for age, gender, and pregnancy. For each step, the least significant variable was removed from the model, until all variables in the model reached significance (*P* < 0.05) and the model was significant.

## Results

A total of 134 case sera were sent from 12 laboratories during the inclusion period.No sera were received from two laboratories. High avidity was the main reason for exclusion (34/38; 90%), and IgM positivity could not be confirmed in the other four cases. Sera from 96 cases met the inclusion criteria for acute disease, of which 26 cases were from the northern part of the Netherlands, 12 cases from the eastern part, 20 from the western part, and 38 from the southern part. A questionnaire was available for 48 cases. Men were less likely to complete the questionnaire (33%; 13/40) than women (66%; 35/53; *P* = 0.001), gender of three cases without a questionnaire was unknown. Median age of non-respondents was significantly lower (28 years; 4–62 years) compared to 37.5 years (7–69 years) of respondents (*P* = 0.013). Completion of the questionnaire was not related to the year of inclusion (*P* = 0.74). In addition to the 48 cases, questionnaires were also received from 50 controls. Ninety percent of the cases and 72% of the controls were tested because of health complaints or symptoms (Table 1). Other reasons for testing included general medical examination/ request by a physician, pregnancy without health complaints, and previous or occupational contact with cats. Most reported symptoms on the 9 months before testing were fatigue and swollen lymph nodes in both cases (73% and 71%, respectively) and controls (64% and 54%, respectively). The only significant differences in pretest symptoms were stomach pain and skin rash, both of which were reported more frequently by the controls.

**Table 1.** Demographic characteristics, reason of testing and symptoms, for cases of acute toxoplasmosis and controls with a completed questionnaire

Variables categories	Cases *n* (%)	Controls *n* (%)	*P*-value*
*N*	48	50	
Gender			
male	13 (27)	19 (38)	0.25
female	35 (73)	31 (62)	
pregnant	3	10	
Age (years)			
median (range)	37.5 (7–69)	31.5 (2–70)	0.09
<18	4 (8)	7 (14)	0.36
18–29	12 (25)	13 (26)	
30–39	11 (23)	15 (30)	
40–49	9 (19)	10 (20)	
>49	12 (25)	5 (10)	
Reason of testing			
Health reasons	43 (90)	36 (72)	0.07
Other reasons	4 (8)	13 (26)	
Medical examination/request of doctor	2	5	
Pregnancy without health complaints	1	5	
Previous/work-related contact with cats	1	2	
Unknown	0	1	
Unknown	1 (2)	1 (2)	
Symptoms in 9 months before testing			
Fever	17 (35)	13 (26)	0.31
Fatigue	35 (73)	32 (64)	0.34
Swollen lymph nodes	34 (71)	27 (54)	0.09
Headache	19 (40)	23 (46)	0.52
Myalgia	15 (31)	12 (24)	0.42
Stomach ache	4 (8)	18 (36)	0.001
Sore throat	7 (15)	11 (22)	0.34
Skin rash	4 (8)	12 (24)	0.04

* *P*-values are from chi-square tests.

Four cases and seven controls were younger than 18 years. These low numbers did not allow separate analyses of children. However, inclusion of children in the adult analyses was also not opportune due to expected differences in eating habits, such as consumption of raw or undercooked meat, and hygiene habits, especially among younger children. This left 44 cases aged 18–69 years and 43 controls aged 19–70 years, of whom 13 women (three cases and 10 controls) were pregnant. Table 2 shows the risk factors identified in the univariable analysis. Beef and veal were consumed more frequently by the cases than by the controls, both in general and in the case of raw/undercooked beef. If split into the consumption of specific beef products, beef that was prepared undercooked (e.g., steak), steak tartare, and roast beef were risk factors for acquiring toxoplasmosis. Consumption of lamb, duck/goose, and big game animals was also more often reported by cases. Consumption of pork was a risk factor for toxoplasmosis only if it was prepared undercooked, or prepared as raw bacon, spreadable sausages, or toppings made from raw pork. Furthermore, consumption of raw or undercooked crustaceans or shellfish was also reported more often by cases. Finally, hand washing before food preparation was found to be a risk factor. Recreation in wooded areas was reported more often by controls.

**Table 2.** Odds ratios of exposures mentioned by at least 20% of the cases and *P* < 0.15 (univariable logistic analyses) in the case control study on acute toxoplasmosis

Variables categories	Cases *n* (%)	Controls *n* (%)	OR (95% CI)*
*N*	44	43	
Consumption of meat			
Beef	43 (98)	34 (79)	**11.4 (1.4–94.3)**
Veal	16 (36)	4 (9)	**5.6 (1.7–18.5)**
Lamb	18 (41)	7 (16)	**3.6 (1.3–9.8)**
Duck/goose (farm)	12 (27)	3 (7)	**5.0 (1.3–19.3)**
Large game animals	14 (32)	3 (7)	**6.2 (1.6–23.6)**
Consumption of raw/undercooked meat			
Any raw/undercooked beef	40 (91)	29 (67)	**4.8 (1.4–16.2)**
Beef prepared undercooked	31 (70)	16 (37)	**4.0 (1.6–9.9)**
(Steak) tartare	18 (41)	9 (21)	**2.6 (1.0–6.8)**
Carpaccio	23 (52)	15 (35)	2.0 (0.9–4.8)
Roast beef	19 (43)	9 (21)	**2.9 (1.1–7.4)**
Smoked beef	16 (36)	9 (21)	2.2 (0.8–5.6)
Any raw/undercooked pork	35 (80)	28 (65)	2.1 (0.8–5.5)
Pork prepared undercooked	17 (39)	5 (12)	**4.8 (1.6–14.6)**
Raw bacon	18 (41)	8 (19)	**3.0 (1.1–8.0)**
Spreadable pork sausage	21 (48)	11 (26)	**2.7 (1.1–6.6)**
Toppings of raw pork	9 (20)	2 (5)	**5.3 (1.1–26.0)**
Dried/smoked sausage	26 (59)	18 (42)	2.0 (0.9–4.7)
Consumption of raw/undercooked crustaceans or shellfish	10 (23)	2 (5)	**6.0 (1.2–29.4)**
Consumption of fresh fruit or vegetable juice	23 (52)	15 (35)	2.0 (0.9–4.8)
Hand washing before preparing food			
Always	15 (34)	28 (65)	1.0
Sometimes	19 (43)	13 (30)	**2.7 (1.1–7.0)**
Never/not applicable	10 (23)	2 (5)	**9.3 (1.8–48.2)**
Contact with sand/soil			
Never	7 (16)	8 (19)	1.0
Monthly	19 (43)	28 (65)	0.8 (0.2–2.5)
Weekly	18 (41)	7 (16)	2.9 (0.8–11.2)
Animal contact (profession, volunteer work)	13 (30)	7 (16)	2.2 (0.77–6.1)
Recreation in wooded area	28 (64)	38 (88)	**0.2 (0.1–0.7)**

* Odds ratios (OR) with 95% confidence interval (95% CI) shown in bold are significant (*P* < 0.05).

After adjustment for age, gender, and pregnancy, two factors remained as risk factors in the final model: consumption of meat of large game animals (adjusted OR: 8.2; 95%CI 1.6–41.9) and washing hands occasionally or never before preparing food (aOR 4.1; 1.1–15.3 and 15.9; 2.2–115.5, respectively). Recreation in wooded areas remained to be associated with a lower risk of being infected by *T. gondii* (aOR 0.1; 0.03–0.6).

## Discussion

Risk factors for *T. gondii* infection are mainly studied in retrospective studies based on IgG seropositivity. In the Netherlands, the three serosurveys done in 1996/1997 [7], 2006/2007 [8], and 2016/2017 [unpublished observations] were used to also analyze risk factors for anti-*T. gondii* IgG antibodies in the general population. In such serosurveys, the moment of infection cannot be taken into account as this is unknown, and associations between exposure and infection do not necessarily indicate a causal relationship. Consumption of various meat products formed the largest group of risk factors for recent *T. gondii* infection in the current univariable analyses, of which consumption of large game such as deer and wild boar remained a risk factor in the multivariable model. The other risk factor in the multivariable model was inadequate hand washing before food preparation Consumption of meat products has been described as a source of outbreaks [6] and as a risk factor in other case–control studies [11–16]. In a study in England and Wales, consumption of beef, especially when consumed raw or undercooked, was most strongly associated with recent *T. gondii* infection [11]. Consumption of raw ground beef, rare lamb, or locally produced cured, dried or smoked meat remained the meat products associated with recently acquired toxoplasmosis in the multivariable analysis of a study in the United States [12]. Meat products previously reported to be associated with recent infections in pregnant women were raw or undercooked lamb, mutton, beef, pork, and game, raw or undercooked minced meat products, and cured pork [13–16]. As cats are essential within the life cycle of *T. gondii* as they shed oocysts in the environment, it was hypothesized that having a cat or having contact with cats or its excrements are risk factors. Yet, only a few studies reported cat-related risk factors: having three or more kittens [12], having a pet cat [13], or cleaning the cat litter box [16]. In our study, none of the variables about cats or its excrements appeared to be associated with a recent *T. gondii* infection. Possibly, since cats shed oocysts for only 2 to 3 weeks during primary infection, whereas oocysts can survive in the environment for months, other routes of exposure to oocysts may be more important than direct contact with cats. Hand hygiene was included in two studies, in which this topic was inquired as “wash hands after food preparation and/or wash hands before meals” [13] or “wash hands after handling raw meat” [12]. In both studies, these factors did not reach significance. However, in our study, washing hands before food preparation was associated with toxoplasmosis, whereas hand washing after preparing or cutting up raw meat or after contact with soil or sand was not. This suggests that infection could also occur through cross-contamination of food via sources other than food. Other risk factors identified in previous studies, but not in our study were working with meat, drinking unpasteurized goat milk, eating raw oysters, clams, or mussels [12], consumption of raw vegetables eaten outside the home [13], contact with soil, travel outside Europe and the United States [14], consumption of unwashed raw vegetables or fruits, and irregular washing of knives after preparing raw meat before handling another food [16]. Milk and dairy products, water, and raw vegetables have also been reported as sources of toxoplasmosis outbreaks [6]. Recreation in wooded areas showed a lower chance of having a recent infection in our study. The potential underlying mechanism is unclear.

In the Netherlands, source attribution of *T. gondii* infections is also carried out using quantitative microbial risk assessment (QMRA). Note that sources identified as important at the population level may do not necessarily be associated with a high odds ratio in a case–control study as odds ratios do not take into account the prevalence of exposure in the population. Both the original and updated QMRA for meatborne infections suggest that filet americain (a raw beef spread) is the most important source in the Dutch population [21, 22]. Filet americain was not identified as a risk factor in the current study, while game meat was not included in the QMRA model. Although many knowledge gaps and uncertainties were identified in the QMRA model for *T. gondii* infections, the results indicated that transmission via soil may be more important than anticipated [9]. This is in contrast to the current study as, with the exception of consumption of raw crustaceans and shellfish, factors related to meat consumption were mainly identified as risk factors in the univariable analyzes.

An important limitation of our study is the small study population. The inclusion of both cases and controls was lower than anticipated at the start of the study. The number of cases identified at the laboratories was lower than estimated in advance based on the seroprevalence of *T. gondii* antibodies in the Netherlands. The numbers dropped even further during the COVID-19 pandemic (12 of the 96 cases were included in 2020–2021; data not shown), indicating that not all cases are identified or included by the laboratories. Among the identified cases with acute toxoplasmosis, only 50% actively participated by completing the questionnaire. Cases and controls were contacted through two intermediaries: RIVM sent the materials to the laboratories, which then asked physicians to send the materials to the cases and controls. Therefore, it is unclear how much each part of this chain contributed to the non-response rate. Laboratories that contacted submitting physicians to increase participation occasionally received feedback that the physician had chosen not to send the questionnaire to a patient, especially in case of perceived personal suffering of the patient. The study aimed to invite three controls per case to end up with at least one control per case. Although about as much cases as controls were included in the analyses, matching was not possible as a large part of the participating controls were invited on the basis of acute toxoplasmosis cases that did not respond. The greatest impact of the small study size was seen in children as only four cases and seven controls under the age of 18 years could be included. This made it impossible to analyze potential risk factors for this age group. Separate analyses for pregnant women were also not possible due to the small numbers, but they were included in the overall analyses with pregnancy as a confounding factor. Nevertheless, no statements on specific risk factors for pregnant women can be given, which is unfortunate as they are a specific risk group for toxoplasmosis and may have altered their exposure to known risks for *T. gondii* infection.

The risk factors presented are based on habits and preferences in the 9 months before the test and not on specific moments of exposure. The long reference period may have introduced recall bias. However, habits and preferences are generally stable over a period of time, reinforcing the reliability of the answers, especially as it addresses the recent past and refers to the period adjacent to the infection.

In conclusion, studies about risk factors for recent *T. gondii* infection are scarce, and most point towards consumption of raw or undercooked meat as an important factor. However, the type of meats varies between studies. In our study, various types of meat came out of the univariable analyses, but only consumption of game meat still proved significant in the multivariable analysis. Vulnerable persons such as pregnant women should therefore continue to be advised to eat only thoroughly cooked meat [23]. Although the evidence for the effect of hand washing is less clear, it also appears to be prudent, especially before preparing food.

## Data Availability

The data that support the findings of this study is available from the corresponding author on reasonable request, with exception of data that could violate the privacy of research participants.
